# Lipid Interaction and Membrane Perturbation of Human Islet Amyloid Polypeptide Monomer and Dimer by Molecular Dynamics Simulations

**DOI:** 10.1371/journal.pone.0038191

**Published:** 2012-05-31

**Authors:** Yun Zhang, Yin Luo, Yonghua Deng, Yuguang Mu, Guanghong Wei

**Affiliations:** 1 State Key Laboratory of Surface Physics, Key Laboratory for Computational Physical Sciences (MOE), and Department of Physics, Fudan University, Shanghai, China; 2 School of Biological Sciences, Nanyang Technological University, Singapore, Singapore; University of Akron, United States of America

## Abstract

The aggregation of human islet amyloid polypeptide (hIAPP or amylin) is associated with the pathogenesis of type 2 diabetes mellitus. Increasing evidence suggests that the interaction of hIAPP with β-cell membranes plays a crucial role in cytotoxicity. However, the hIAPP-lipid interaction and subsequent membrane perturbation is not well understood at atomic level. In this study, as a first step to gain insight into the mechanism of hIAPP-induced cytotoxicity, we have investigated the detailed interactions of hIAPP monomer and dimer with anionic palmitoyloleolyophosphatidylglycerol (POPG) bilayer using all-atom molecular dynamics (MD) simulations. Multiple MD simulations have been performed by employing the initial configurations where the N-terminal region of hIAPP is pre-inserted in POPG bilayer. Our simulations show that electrostatic interaction between hIAPP and POPG bilayer plays a major role in peptide-lipid interaction. In particular, the N-terminal positively-charged residues Lys1 and Arg11 make a dominant contribution to the interaction. During peptide-lipid interaction process, peptide dimerization occurs mostly through the C-terminal 20–37 region containing the amyloidogenic 20–29-residue segment. Membrane-bound hIAPP dimers display a pronounced ability of membrane perturbation than monomers. The higher bilayer perturbation propensity of hIAPP dimer likely results from the cooperativity of the peptide-peptide interaction (or peptide aggregation). This study provides insight into the hIAPP-membrane interaction and the molecular mechanism of membrane disruption by hIAPP oligomers.

## Introduction

Many human diseases such as Alzheimer's, type II diabetes, and Parkinson's are pathologically characterized by the formation of protein fibrillar deposits. For each disease, a specific protein is involved in amyloid formation. The fibrillization is generally described by a nucleation-dependent polymerization process characterized by a lag phase associated with the formation of a nucleus, after which fibril elongation occurs rapidly [1]. Although the molecular mechanism behind the cytotoxicity is poorly understood, increasing evidence suggests that small oligomers formed in the earlier stage of aggregation are the main cytotoxic species and the interaction of these oligomeric species with biological membranes may cause the loss of membrane integrity [2], [3].

In type II diabetes mellitus, the major component of amyloid deposits is human islet amyloid polypeptide (hIAPP or amylin), a 37-residue peptide hormone co-secreted with insulin by the islet β-cells of pancreas. Approximately 95% of all patients with type II diabetes mellitus have large extracellular deposits composed of fibrillar hIAPP. The hIAPP found in amyloid plaques is wild type, containing a disulfide bond between Cys2 and Cys7 and having an amidated C-terminus [4]. It has been proposed that hIAPP can cause significant impairment of the integrity of the phospholipid membrane in both model membranes and in cells [5], [6]. Current models of hIAPP-induced membrane disruption suggest that either aggregation intermediates (e.g., oligomers) are the most toxic species or the process of fibrillization is the primary cause of membrane disruption [1], [7], [8]. The exact mechanism of membrane disruption is unknown but has been linked to peptide aggregation on the membrane surface [1], [9], [10].

Previous in vitro studies have shown that hIAPP can readily bind to lipid bilayers and this binding can accelerate hIAPP aggregation and induce subsequent membrane disruption [9], [11]–[14]. The monomeric and oligomeric hIAPPs have been reported to adopt predominantly helical structure in the presence of negatively-charged membrane environment [9], [12], [14]–[17] and the latter state most strongly correlates to membrane damage [9], [18]. Despite extensive experimental studies, an understanding of the first step of hIAPP-membrane interaction at atomic level is still missing.

Recent computational studies have focused on the structures of monomeric and oligomeric species of full-length hIAPP [19]–[23] and of its amyloidogenic fragments [24]–[28] (such as residues 22–27 and 20–29) using coarse-grained or all-atom protein model, however, they are mainly conducted in aqueous solution without lipid bilayers. There are only a few studies performed in the presence of a few of lipid molecules or bilayers for hIAPP fragments [27], [28]. To our knowledge, this is the first computational study to investigate the detailed interaction of full-length hIAPP monomer/dimer with negatively charged palmitoyloleolyophosphatidylglycerol (POPG) lipid bilayer and subsequent membrane perturbation. In this study, lipid interaction and bilayer perturbation of hIAPP monomer and dimer will be explored by performing all-atom molecular dynamics (MD) simulations in a fully solvated explicit POPG lipid bilayer.

## Materials and Methods

The amino acid sequence of hIAPP is KCNTATCATQ^10^RLANFLVHSS^20^NNFGA ILSST^30^NVGSNTY, with the Cys2 and Cys7 forming a disulfide bond. To mimic the experimental neutral pH condition, the side chains of Lys1 (Lys+) and Arg11 (Arg+) are charged. The N-terminus is also charged (NH_3_+), while the C-terminus is amidated. The model membrane consists of 2×64 POPG lipids (i.e., 64 lipids in each leaflet) and the initial coordinates are obtained from a previous computational study of a pure POPG lipid bilayer by Elmore et al. [29]. Counterions (Na+) are added to neutralize the system.

Three different systems are studied, which are labeled as 1mono, dimer (including dimer1, dimer2, and dimer3), and 2mono according to the number of hIAPP peptide chains included as well as the distance between two chains (see below for more details). Previous studies reported that hIAPP peptides partitioned into monomeric and oligomeric helical assemblies in the presence of negatively-charged SDS micelle or lipid membrane, with their N-termini oriented towards the membrane [11], [30]. As computational studies of spontaneous peptide partitioning into atomic detail lipid bilayers are thought to be unfeasible due to long simulation time scales required to capture insertion events at physiological temperatures [31], the starting states for MD simulations are membrane-bound helical hIAPP with the N-terminal residues 1–14 pre-inserted inside the upper leaflet of a POPG lipid bilayer, as done recently by us for the Alzheimers amyloid beta 25–35 fragment [32]. The distance from the centroid of residue Lys1 to the bilayer surface is 1.5 nm. The lipids overlapping with the peptide are adjusted through energy minimization and then removed if the overlap still exists after energy minimization, which could be done by the program INFLATEGRO from P. Tieleman's group [33]. Each system is simulated in the presence of explicit water and counterions.

### 1mono system

**Figure 1:**
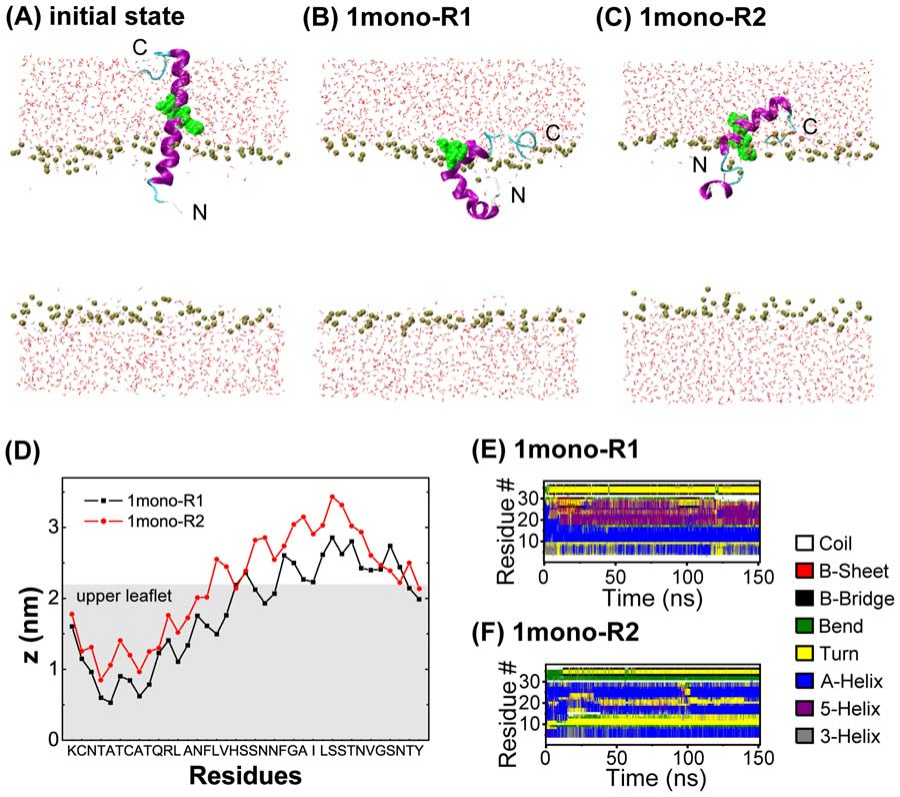
Initial state and the simulation results of 1mono system in two independent MD trajectories. (A): initial state. Final states (t = 150 ns) generated in MD runs of 1mono-R1 (B) and 1mono-R2 (C); The z-position of each amino acid residue represented by the z-position of the atom with the smallest z-coordinate (D). The time evolution of hIAPP secondary structure in MD runs of 1mono-R1 (E) and 1mono-R2 (F). The z-coordinate of the bilayer center is zero. In all the snapshots, peptide helical structure is in purple and other secondary structures are in cyan, water molecules are in red dots, and the phosphorus atoms of lipids are in tan spheres. Residues 18–20 are in green and in vdW representation. For clarity, counterions and the other atoms of POPG lipids are not shown.

This system contains one hIAPP peptide chain with the N-terminal residues 1–14 pre-inserted inside a POPG lipid bilayer (see Fig. 1(A)). This allows us to explore the detailed interactions of monomeric hIAPP with a POPG lipid bilayer. The initial conformation of hIAPP is an NMR-derived structure (pdb ID: 2KB8) solved in sodium dodecyl sulfate (SDS) micelles. The core of the structure is an α-helix running from residues 5–28 with a distortion or kink near residues 18–22 [17]. This distortion introduces pliancy in the angle between the N- and C-terminal segments of the α-helix.

### Dimer system

**Figure 2:**
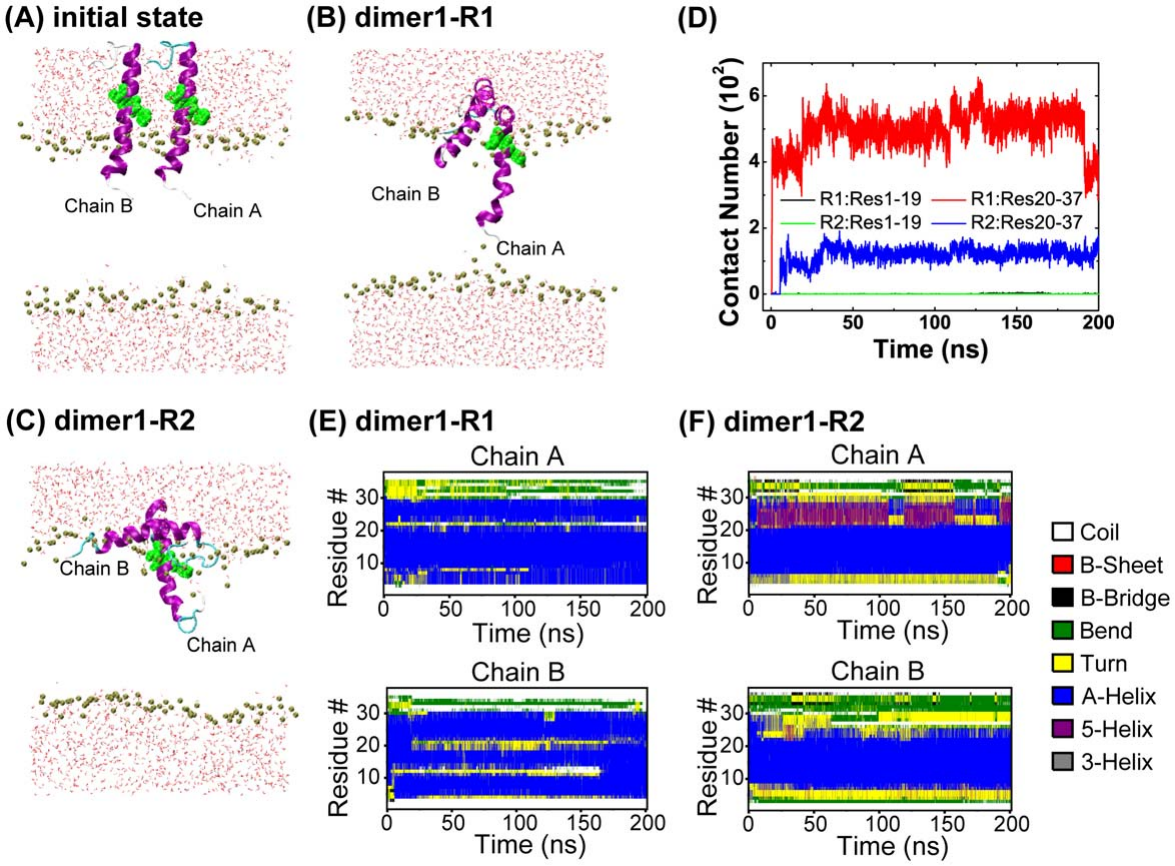
Initial state and the simulation results of dimer1 system in two independent MD trajectories. (A): initial state. The final states (t = 200 ns) generated in dimer-R1 (B) and dimer-R2 (C); time evolution of the number of atomic contacts between two different regions (residues 1–19 and 20–37) of chain A and chain B (D) and the secondary structure profile of hIAPP in MD runs of dimer-R1 (E) and dimer-R2 (F). Snapshots (A)∼(C) are shown by using the same representations as those used in Fig. 1.

This system contains two identical hIAPP peptide chains (see Fig. 2(A)) with each chain having the same conformation as that in 1mono system. The center-of-mass distance between the two chains is 2.1 nm. The minimum distance between the two chains is 0.8 nm, i.e. free of any inter-chain atomic contacts, but with weak inter-peptide interaction. This dimer is labelled as dimer1. The choice of this system is used to study peptide-lipid interaction and the effect of hIAPP peptide-peptide interaction on membrane perturbation. To examine whether dimer interfaces affect peptide-lipid interaction and peptide aggregation, another two dimers are constructed by rotating each hIAPP chain in dimer1 90° around its backbones under clockwise or counter-clockwise directions (see Fig. S1 for more details). The two dimers are labelled as dimer2 and dimer3, with a center-of-mass distance of 2.1∼2.2 nm between the two chains.

### 2mono system

This system contains two identical hIAPP peptide chains with each chain having the same conformation as that in dimer1 system. The two chains are separated by a minimum distance of 2.5 nm, i.e. free of any inter-chain contacts (see Fig. S5(A)). The two hIAPP peptides in this system can be considered as two monomers. The choice of this system is used to examine whether increased monomer concentrations and/or peptide-peptide interactions play a crucial role on bilayer perturbation.

### MD simulations

All the MD simulations are performed in the isothermal-isobaric (NPT) ensemble using the GROMACS 3.3.3 software package [34]. The POPG lipid parameters are the same as that used in Elmore's work [29], a modified version of Berger force field [35] with an adaption of Tieleman's lipid parameters (http://moose.bio.ucalgary.ca/). The rest parts of the system are described with the GROMOS-87 force field [36]. The water is modelled by the simple point charge (SPC) model [37]. Bond lengths of peptides and lipids are constrained with LINCS [38] and water geometries are constrained with SETTLE [39], which allow an integration time step of 2 fs. Long-range electrostatic interaction is calculated using the Particle Mesh Ewald (PME) method [40] with a real space cutoff of 1.2 nm, as recommended for membrane simulations, especially for those involving charged lipids [41]. The van der Waals interaction is calculated using a cutoff of 1.4 nm. The temperature of the system is maintained close to 310 K, above the gel-liquid crystal phase transition temperature (271 K) of the POPG lipid membrane [42], as done recently by Tolokh et al. [43]. Lipids, water, peptide, and counterions are separately coupled to the temperature bath by weak coupling with a coupling constant of 0.1 ps [44]. The pressure is also weakly coupled (with a coupling constant of 1.0 ps and a compressibility of 4.5×10^−5^ bar^−1^) [44] using anisotropic scheme in which the pressures in x, y, z directions are coupled to 1.0 bar separately, as Elmore did in the MD simulation of a pure POPG lipid bilayer [29]. All MD simulations are performed using periodic boundary conditions in a rectangular box with a size of 5 nm×7 nm×9 nm. Two independent 150- or 200-ns MD runs are carried out for each system using different initial velocity distributions, starting from the same initial state, except when mentioned otherwise. A summary of the MD setup details is given in Table 1.

**Table 1:** Set up details of all MD simulations at 310 K.

system	name of MD run	number of lipid molecules	number of water molecules	simulation time (ns)	initial state
1mono	1mono-R1	128	4393	150	Fig. 1(A)
	1mono-R2	128	4393	150	Fig. 1(A)
dimer1	dimer1-R1	127	4798	200	Fig. 2(A)
	dimer1-R2	127	4798	200	Fig. 2(A)
dimer2	dimer2	127	4771	200	Fig. S4(A)
dimer3	dimer3	127	4759	200	Fig. S4(D)
2mono	2mono-R1	126	4810	150	Fig. S6(A)
	2mono-R2	126	4810	150	Fig. S6(A)
POPG	POPG	128	5145	100	—

For each system, we describe the name of the system, the name of MD runs, the number of lipid and water molecules, the simulation time and the initial state of each MD run. To mimic the experimental neutral pH condition, the side-chains of Lys (Lys+), Arg (Arg+) and N-terminus (NH3+) are all charged. The C-terminus is amidated. Counterions (Na+) are added to neutralize the system.

#### Analysis

We perform the analysis using our in-house-developed codes and the GROMACS facilities. The position of each amino acid residue in water/bilayer is described by the z-position of the atom with the smallest z-coordinate. The insertion depth of hIAPP peptide is estimated by the z-position of the most deeply inserted residue in the bilayer. The secondary structure profile is given by the DSSP program [45]. In the dimer system, the inter-peptide interaction of the N-terminal residues 1–19 and that of the C-terminal residues 20–37 are estimated by the number of atomic contacts. Here an atomic contact is defined when two nonhydrogen atoms come within 0.54 nm. To examine the effect of hIAPP on the ordering of bilayer surface, we calculate the root-mean-square displacement of z-position of phosphorus atom (z-displacement) in the POPG head group for each leaflet. The bilayer thickness distribution over the x-y lateral plane (with a size of 5 nm×7 nm) is calculated for each system. In the calculation, the thickness of bilayer is estimated by the local average of phosphor-to-phosphor distance [29]. All the systems are displayed using the VMD program [46].

## Results

### hIAPP monomer has a preference to bind near the surface of POPG bilayer and keeps mostly α-helical structure

To examine the detailed interactions of hIAPP monomer with a POPG bilayer, we have performed two independent 150-ns MD runs on 1mono system. The two runs are labeled as 1mono-R1 and 1mono-R2. The initial and final (t = 150 ns) states, the smallest z-position of each residue in bilayer, together with the secondary structure profiles of hIAPP, are given in Fig. 1. In order to see the insertion depth of hIAPP, residues 18–20 are in green and in vdW representation in Fig. 1(A)–(C). In the initial configuration, the N-terminal residues Lys1∼Asn14 are embedded in the POPG bilayer and residues 15–37 are exposed to the aqueous phase (see Fig. 1(A)). It can be seen from Fig. 1(B), (C), (D) that in the final state three more residues F15-L16-V17 insert into the bilayer while residues 20–37 lie on the bilayer surface. This makes the whole hIAPP peptide stay near the bilayer surface with the N-terminal 1–19 residues binding to the upper leaflet of the bilayer. Membrane binding perturbs the integrity of the helix. A helix kink, which locates at the head group region of the bilayer, appears around residues 18–20. This allows an angle forming between the N- and C-terminal segments of the α-helix. Although the N-terminal residues 1–17 are inside the bilayer, residues 1–5 bend upward and Lys1 anchors to the headgroup of the upper leaflet, which is probably due to strong electrostatic attraction between the positively charged Lys1 and the negatively charged head group of POPG (see below for more detailed discussion). The time evolution of the secondary structure profiles of hIAPP in the two trajectories are given in Fig. 1(E) and (F). We see that the region spanning residues 5–28 mostly keeps helical structure during the full period of the 150 ns simulation, albeit with some structure distortions in the region of residues 10–20. These results are in good agreement with previous experimental studies reporting that the membrane-bound hIAPP monomer is predominantly α-helical in structure [15] and the membrane binding site is largely localized to the N-terminal 1–19 residues of the peptide [30].

### hIAPP peptide in dimeric state inserts deep into POPG bilayer or anchors to bilayer surface depending on dimer interface

Having now established that the force-field used for the membrane-bound hIAPP monomer yields a helical conformation in good agreement with the experimentally-generated structure in membrane, we turn to study the interaction behavior of dimeric hIAPP with a POPG bilayer using the same peptide/lipid force field. To this aim, we have performed two 200-ns MD simulations on dimer1 system starting from the initial state shown in Fig. 2(A) using different initial velocity distributions. The two MD runs are labeled as dimer1-R1 and dimer1-R2. More details about the dimer interface can be seen from Fig. S1. Figure 2(B) and (C) presents the final configurations (at t = 200 ns) of the system obtained in the two MD runs. We see that chain A inserts more deeply with residues 1–21 buried inside the POPG bilayer while chain B binds to the bilayer surface. For chain B, residues 1–15 are buried inside the bilayer whereas residues 20–37 are exposed to the aqueous environment. Of interest, it is observed in run dimer-R1 that the N-terminal residue Lys1 in chain A anchors to the lipid headgroup of the lower leaflet (see Fig. 2(B)) and the chain traverses the membrane bilayer. In run dimer-R2, the N-terminal residues 1–5 of chain A bend upward and Lys1 binds to the headgroup of the upper leaflet, displaying a similar binding behavior as that observed in the two trajectories of 1mono system. By comparing the snapshots in Fig. 2(B)–(C) with those in Fig. 1(B)–(C), we see that hIAPP peptide in dimer system can insert more deeply into the bilayer than hIAPP monomer. It is noted that during the membrane insertion process, the helical structure of hIAPP is mostly preserved, as seen from the time evolution of the secondary structure profile shown in Fig. 2(E) and (F)).

**Figure 3:**
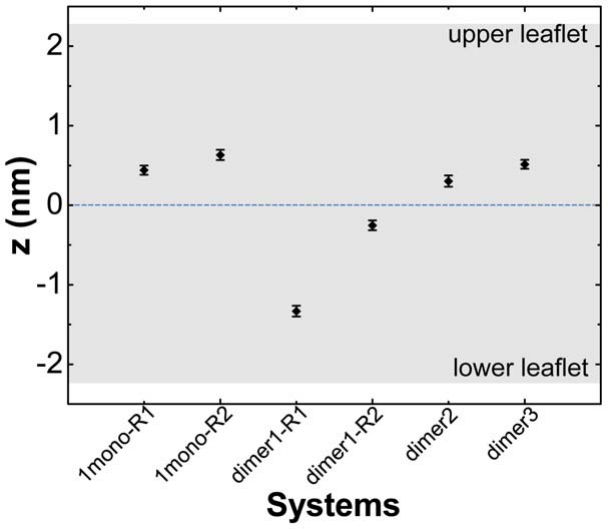
Bilayer insertion depth of hIAPP in monomeric and dimeric states. The insertion depth of hIAPP peptide is estimated by the z-position of the most deeply inserted residue in the bilayer. The z-position is an average of the last 50 ns of each MD run. The z-axis is normal to the bilayer surface. The z-coordinate of the bilayer center is zero. Upper leaflet: z>0 nm, lower leaflet: z<0 nm.

To quantitatively compare the peptide insertion depth in the two different systems, we plot in Fig. 3 the time-averaged z-coordinate of the peptide atom inserted most deeply in the bilayer over the last 50 ns for all the MD runs of 1mono and dimer1 systems. It can be seen that in the two MD runs of 1mono system, the deepest part of hIAPP peptide is still located in the upper leaflet, while in the MD runs of dimer1 system it is in the lower leaflet. This indicates that hIAPP peptide in dimer1 system inserts more deeply in the POPG lipid bilayer than that in 1mono system. As the membrane insertion process of hIAPP in dimer system is accompanied by the inter-peptide interaction between C-terminal residues 20–37 (see Fig. 2(D)), the inter-peptide interaction likely facilitates membrane insertion. To make certain that the higher insertion propensity of hIAPP in dimeric state is an intrinsic character of hIAPP peptide rather than the stochastic output of MD simulations, we have performed more MD simulations. In the four 150-/200-ns long-time MD simulations, the final location of the peptide, which is either in the upper leaflet or in the lower leaflet can be roughly estimated in the first 10 ns although the process of membrane insertion is not finished yet and the insertion depth may change with the increase of simulation time (see Fig. S2). Therefore, another eight short-time (10 ns) MD simulations are conducted using different initial velocity distributions starting from the state of Fig. 2(A). For comparison, eight independent 10-ns MD runs have also been performed for 1mono system starting from the state of Fig. 1(A). The maximum insertion depths of hIAPP in the 16 MD runs are given in Fig. S3. It can be seen that hIAPP peptide in three out of eight MD runs of dimer1 system inserts into the lower leaflet of the bilayer, whereas peptide in the eight MD runs of 1mono system binds to the upper leaflet.

To examine whether dimer interfaces affect the insertion depth of hIAPP in POPG bilayer, another two dimer systems (dimer2 and dimer3) are constructed by rotating each hIAPP chain in dimer1 90° around its backbones under clockwise or counter-clockwise directions (see Fig. S1). A 200-ns MD simulation is carried out for dimer2/dimer3 starting from the initial state shown in Fig. S4(A)/(D). The final states (at t = 200 ns) are given in Fig. S4(B) and (E). We see that hIAPP peptides in the two dimers are located in the upper leaflet and keep mostly in helical structure, albeit with different helix-helix orientations. This can also be seen from the z-position of the peptide atom inserted most deeply in the bilayer shown in Fig. 3. Another 18 short-time (10-ns) MD simulations using different initial velocity distributions, with nine runs starting from the state of Fig. S4(A) and the other nine runs starting from the state of Fig. S4(D), show a similar bilayer insertion depth of hIAPP peptide. These results, together with the results from the above four long-time MD runs (1mono-R1, 1mono-R2, dimer-R1, and dimer-R2) of dimer1 suggest that hIAPP in dimer system has a probability to insert deep into POPG bilayer than hIAPP monomer while the insertion depth seems to depend on dimer interfaces.

### Peptide-peptide interaction is mainly through the C-terminal 20–37-residue segment

The detailed information of peptide-peptide interaction during the peptide-lipid interaction process in dimer system can be seen from Fig. 2(D) and Fig. S4(C) and (F). These figures give the time evolution of the number of inter-peptide atomic contacts for residues 1–19 and 20–37. In the four MD runs, the contact number between the N-terminal 1–19 residues of chain A and that of chain B is almost zero during the full period of MD simulations, indicative of neglectable interaction between the N-terminal residues of the two chains. In contrast, the contact number between the C-terminal 20–37 residues increases quickly and reaches to a plateau after 150 ns, although the final contact number is different in the four trajectories. During the process of membrane-bound hIAPP aggregation (dimerization), the helical structure is mostly preserved, as seen from the final state of hIAPP dimer shown in Fig. 2(B) and (C) for dimer1, Fig. S4(B) for dimer2, and Fig. S4(E) for dimer3. This is consistent with experimental observation that the early formed membrane-bound hIAPP oligomers are predominantly α-helical in structure [9]. The data in Fig. 2 and Fig. S4 demonstrate that chain A and chain B interact with each other during bilayer insertion process and form a helical dimer mostly through the C-terminal 20–37 region containing the amyloidogenic 20–29-residue segment independently of dimer interface. Our results support previous experimental hypothesis that residues 20–29 are crucial for membrane-bound hIAPP aggregation [47].

### The interaction of hIAPP peptide with POPG bilayer is mostly driven by the N-terminal positively-charged residues via electrostatic interactions

**Figure 4:**
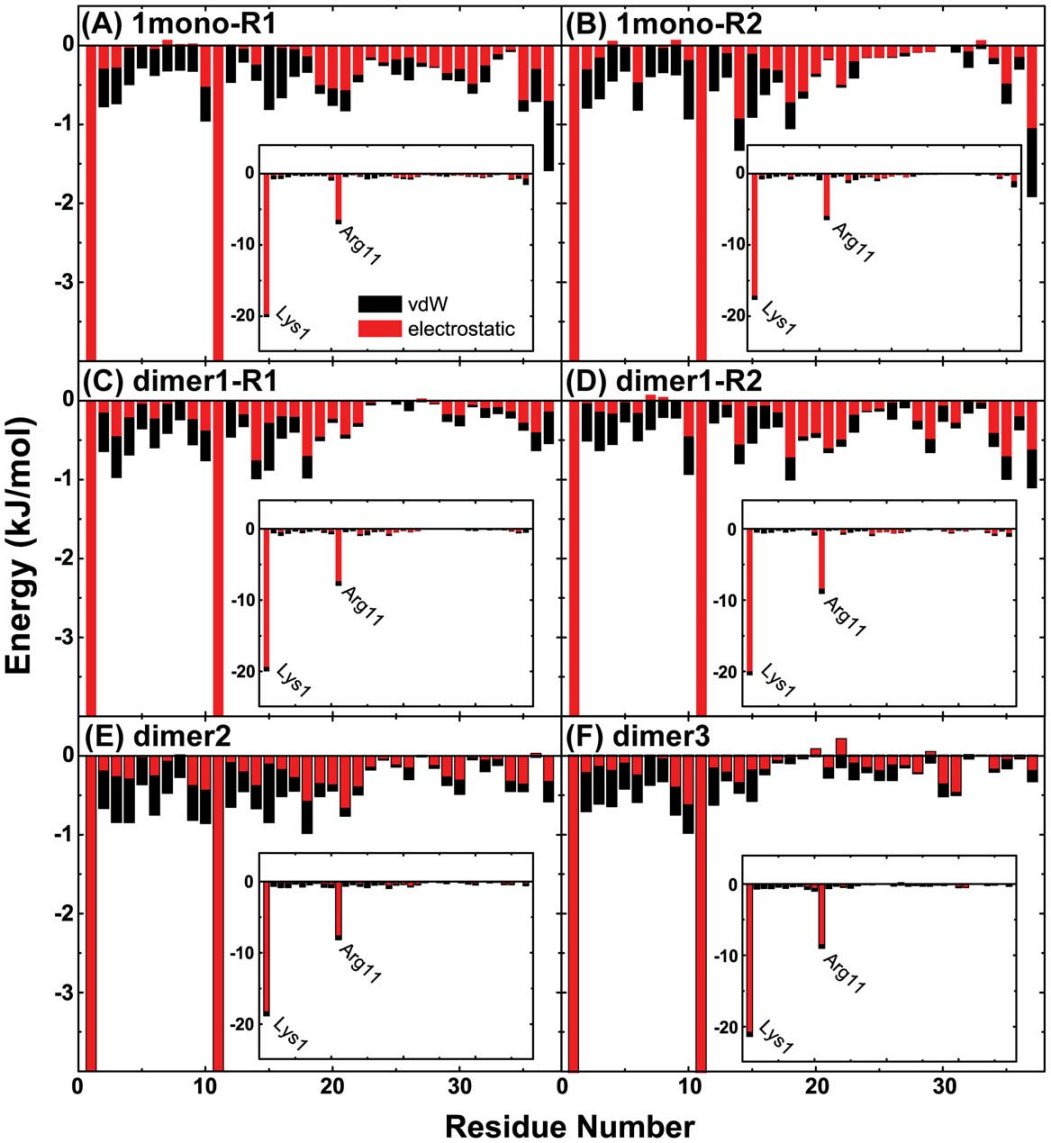
The interaction energy of each individual hIAPP residue with POPG lipid bilayer (per lipid). To show the total interaction energy of Lys1/Arg11 with POPG lipid, the interaction energy using a different scale is given in the inset. The interaction energy is plotted for both monomer and dimer systems and is averaged over the last 50 ns for each MD run. The residue-based interaction energy is decomposed into the electrostatic and vdW terms.

To identify the important residues for hIAPP-POPG interaction, we plot in Fig. 4 the interaction energy of each amino acid residue with the POPG lipid bilayer (per lipid) for all the MD runs of 1mono and dimer systems. To show the total interaction energy of Lys1/Arg11 with POPG lipid, the interaction energy using a different scale is given in the inset. The interaction energy is decomposed into electrostatic and van der Waals (vdW) components. It can be seen from Fig. 4 that for each system electrostatic interaction is much stronger than vdW interaction, indicating that electrostatic interaction plays a dominant role in peptide-lipid interaction. In particular, the positively charged residues of Lys1 and Arg11 have the strongest interactions with the anionic POPG bilayer. Moreover, residue Lys1 at the N-terminus has a much stronger interaction with lipids than Arg11 as it carries 2+ out of the total 3+ charges of hIAPP. This result provides an explanation for the binding behavior observed in the MD runs of 1mono, dimer1, dimer2, and dimer3 systems that the N-terminal residue Lys1 always anchors to the lipid headgroup of either the upper leaflet or the lower leaflet of POPG bilayer (see panels (B) and (C) of Figs. 1 and 2, and panels (B) and (E) of Fig. S4). Our data demonstrate that the interaction of hIAPP monomer/dimer with POPG bilayer is largely driven by the N-terminal residues through electrostatic interaction. Of interest, a membrane-bound α-helical conformation of hIAPP anchoring at or partial insertion into anionic POPG monolayer through interactions with the cationic N-terminal region was reported in a recent infrared reflection absorption spectroscopic study [48].

To examine the lipid group that has strong interactions with hIAPP peptide, we have calculated the interaction energies of peptide-polar head group and peptide-hydrophobic tail group (per lipid). The results are given in Fig. S5 and it is seen from this figure that the interaction energy of peptide-lipid head group is much larger than that of peptide-lipid tail group in both monomer and dimer systems, indicating that hIAPP peptide has a strong interaction with the lipid head group. This result is consistent with the observation in the MD runs of 1mono and dimer (dimer1, dimer2, and dimer3) systems that the N-terminal residue Lys1 always anchors to the lipid head group of POPG bilayer. Overall, our results are consistent with previous experimental studies suggesting that the N-terminal amino acid residues 1–19 are primarily responsible for the interaction of hIAPP with membranes [30], [49].

### hIAPP dimers perturb the POPG bilayer to a larger extent than monomers

**Figure 5:**
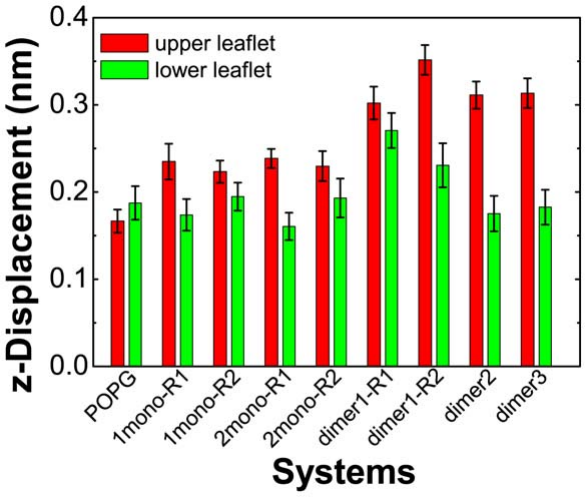
The z-displacement of phosphorus atom in the head group of POPG lipids in each system. The z-displacement of phosphorus atom is calculated for each leaflet. The data are average over the final 50 ns of each MD trajectory.

It has been proposed that hIAPP-membrane interaction disrupts the integrity of the phospholipid membrane and the mechanism of membrane disruption is linked to peptide aggregation on the membrane surface [1], [9], [10]. To probe the impacts of the membrane-bound monomeric/dimeric hIAPP on the ordering of POPG bilayer at atomic level, we first calculate the time-averaged z-displacement of phosphorus atoms relative to the average z-coordinate of all phosphorus atoms in the upper/lower leaflet of bilayer in each frame (see Fig. 5). For comparison, the result from a 100-ns MD simulation of a pure POPG lipid bilayer is also presented. For the lower leaflet, similar z-displacement value is seen for all the systems, except for dimer1-R1 and dimer1-R2 due to hIAPP insertion into the lower leaflet. For the upper leaflet, the z-displacement in 1mono system is slightly larger than that in pure POPG bilayer, whereas the value in dimer system is significantly larger than that in pure POPG. These data indicate that hIAPP dimer has a larger disturbance on the ordering of head groups of POPG bilayer than monomer.

To examine whether the number of membrane-bound hIAPP monomers (or increased monomer concentration) is important for bilayer perturbation, we have conducted two 150-ns MD simulations on 2mono system using different initial velocity distributions starting from the initial state shown in Fig. S6(A). The initial minimum distance between the two chains is 2.5 nm, much larger than that in dimer system. The initial conformations and the initial bilayer insertion depths of hIAPP peptides are the same as those in dimer1 system. The two MD runs are labeled as 2mono-R1 and 2mono-R2. Figure S6(B) and (C) presents the final configurations (at t = 150 ns) obtained in the two MD runs. The insertion depth of each residue is demonstrated by the smallest z-coordinate of the atom that inserts most deeply in the bilayer (Fig. S6(D)). It can be seen that the two chains both bind to the bilayer surface of the upper leaflet and keep mostly α-helical structure. The C-terminal residues 20–37 lie on the bilayer surface and are exposed to the aqueous environment while the N-terminal residues 1–15 stay inside the upper leaflet of the bilayer. The minimum distance between the two chains is larger than 1.4 nm during the last 50-ns MD simulations of the two runs (data not shown), implying that the two peptide chains are in monomeric state. The time-averaged z-displacement of phosphorus atoms relative to the average z-coordinate of all phosphorus atoms in the upper/lower leaflet of each frame is presented in Fig. 5. The z-dispalcement of phosphorus atoms in 2mono system is quite similar to that in 1mono system, much smaller than that in dimer system (for upper leaflet). The distinct difference of z-displacement in 2mono and dimer systems, together with the similarity of z-displacement in 1mono and 2mono systems, suggests that hIAPP peptide-peptide interaction correlates more strongly with the structural disturbance of the POPG head groups than the increased monomer concentration.

**Figure 6:**
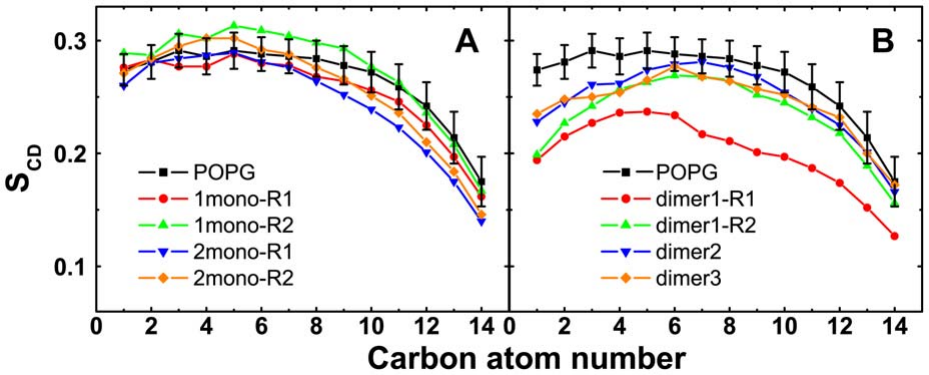
Time-averaged order parameter S_CD_ of *sn*-1 chain of lipid tails in all the systems. The order parameter is averaged over the last 20 ns of each MD trajectory for (A) monomer and (B) dimer systems. In the calculation, the lipids within 1.0 nm from any non-hydrogen atom of hIAPP peptide are considered. For comparison, we also give the S_CD_ of *sn*-1 chain obtained from the last 20 ns of a 100-ns MD run for pure POPG lipid bilayer.

Having elucidated the impact of monomeric/dimeric hIAPP species on the headgroup of POPG bilayer, we then examine their perturbation on the lipid tail by calculating the lipid tail order parameter S_CD_. The S_CD_ value is calculated using the formula S_CD_ = 0.5〈3cos^2^θ−1〉, where, θ represents the angle of the C-H bond vector (in the simulation) or the C-D bond vector (in the experiment) with the bilayer normal. The angular brackets indicate averaging over lipids and over time [50]. Figure 6 gives the local S_CD_ values of acyl chain 1 (*sn*-1) of POPG lipids within 1.0 nm from any non-hydrogen atom of hIAPP peptide in all the MD runs. For comparison, the S_CD_ of *sn*-1 (as well as its error bar) from a 100-ns MD simulation of a pure POPG lipid bilayer is also given. The order parameters of carbon atoms in the lipid tail are quite similar in all the MD runs of pure POPG, 1mono, and 2mono systems (see Fig. 6(A)), whereas a distinct decrease of S_CD_ for carbon atoms 1–4 in the lipid tail heads is observed in the four MD runs of dimer system. This result is consistent with the large z-displacement of phosphorus atoms in dimer system shown in Fig. 5. Remarkably, the S_CD_ values of carbon atoms 5–12 in run dimer-R1 are much smaller than that in other MD runs, indicative of a greater disturbance on lipid tail order by hIAPP in run dimer-R1. Overall, our results suggest that the membrane-bound hIAPP dimer perturbs the lipid tail order of POPG bilayer more strongly than monomer.

**Figure 7:**
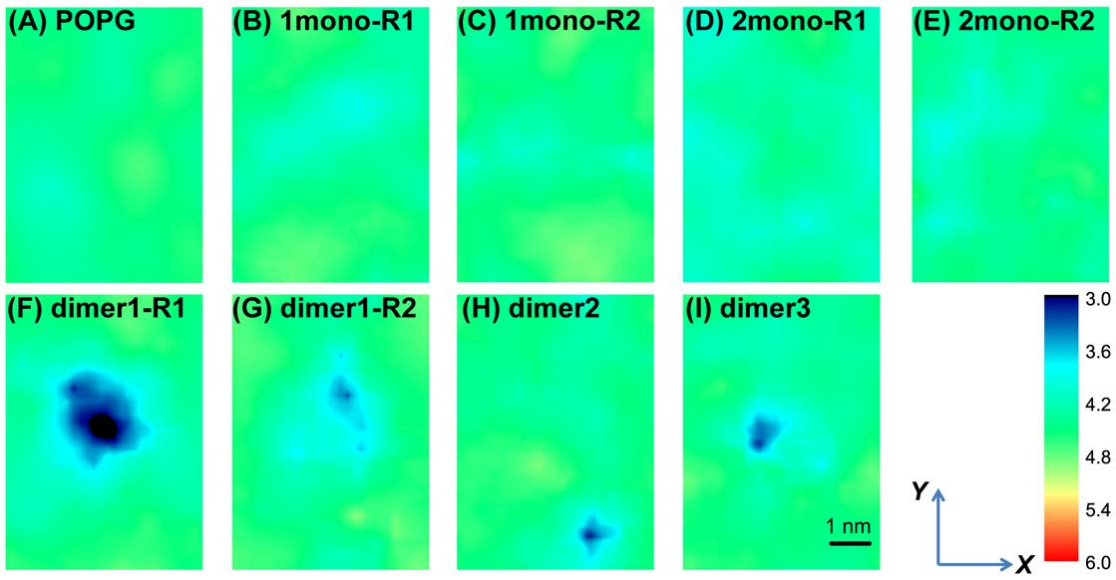
Bilayer thickness distribution map over the x-y lateral plane in each MD run. The x-y lateral plane of each bilayer has a size of 5 nm×7 nm. The centroid of the peptide system in panels (B)∼(I) is in the center of simulation box. The black color in panel (F) indicates that the local thickness of the POPG bilayer is less than 3 nm.

Finally we investigate the influence of membrane-bound hIAPP monomer/dimer on the bilayer thickness. We plot in Fig. 7 the in-plane (the x-y lateral plane of the bilayer) distribution of bilayer thickness in each MD run. It is seen from Fig. 7(A) that pure POPG bilayer is almost uniform with a thickness of ∼4.4 nm. The bilayer thickness in 1mono and 2mono systems is similar to that of pure POPG bilayer, albeit with a larger slightly-thinned area in 2mono system. In contrast, the bilayer in dimer system displays distinct thinning at the position where the hIAPP peptide locates, having a local thickness less than 3.6 nm. These data demonstrate that dimeric hIAPP has a pronounced ability to perturb POPG bilayer than the monomeric species and can lead to membrane disruption. By comparing the data in panels (B)–(C) with those in (D)–(I), we find that the extent of membrane perturbation is affected by the peptide-peptide interaction and the number of membrane-bound hIAPP monomers. However, membrane perturbation is much more sensitive to peptide-peptide interaction (or aggregation) (panels (F), (G), (H), (I)). These findings suggest that membrane disruption is likely cooperative and the cooperativity results from the interaction or aggregation of membrane-bound hIAPP monomers. Interestingly, previous experimental studies suggested that the populations of membrane-bound oligomers are correlated with the capacity of hIAPP to disrupt model membrane [9], [18] and induce cell toxicity [51].

## Discussion

The interactions of several disease-related peptides/proteins (including Aβ, hIAPP, and α-synuclein) with membranes have received considerable attention due to their important role in cytotoxicity [5], [52], [53]. Among these amyloidogenic peptides, hIAPP is one of the most extensively studied systems [8]–[13], [18]. Although membrane-bound oligomers are indicated to be correlated with the capacity of hIAPP to disrupt model membrane [9], [18], little atomic level information exists to show how hIAPP impacts the surrounding lipid matrix and induces membrane disruption. In the present work, lipid interaction and bilayer perturbation of hIAPP monomer and dimer have been investigated by performing multiple 150-/200-ns all-atom MD simulations. Three different systems have been simulated, including 1mono, dimer (dimer1, dimer2, and dimer3), and 2mono. Although we place the hIAPP perpendicular to the plane of POPG membrane in the initial state, the peptide appears to move towards an in-pane orientation and stays near the surface of POPG bilayer in all monomer simulations, consistent with previous NMR studies on hIAPP in membrane environment [14], [15], [17]. Simulations on three different dimers show that dimeric hIAPP has a probability to insert more deeply and transverse POPG bilayer depending on dimer interface. In both cases, the interaction of hIAPP peptide with POPG bilayer is mostly driven by electrostatic interactions. In particular, the N-terminal positively-charged residues Lys1 and Arg11 make a dominant contribution to the interaction, providing direct evidence for previous suggestion that hIAPP binds to POPG monolayer most likely through electrostatic interactions by insertion of its N-terminal part [48]. It is noted that the importance of electrostatic interaction in modulating Aβ-POPC bilayer interactions was also found in a recent MD simulation [54], indicative of some common molecular mechanism behind different amyloidgenic peptide—membrane interaction.

The secondary structure profiles of hIAPP peptide in both monomeric and dimeric states suggest that the membrane-bound hIAPP is mostly in α-helical conformation. To quantitatively display the helix content in monomeric and dimeric hIAPP, we have calculated the percentage of helical structure in monomer (including 1mono and 2mono) and dimer (including dimer1, dimer2, and dimer3) using the data generated in the last 50 ns of 150-/200-ns MD runs. The helix percentages in monomer and dimer systems are 50±4% and 53±9%, respectively, indicative of similar amount of helical structures in the two different systems. Indeed, it has been shown recently that the structure of hIAPP bound to anionic POPS containing vesicles or SDS micelle is an α-helix with residues 9–22 or 5–28 or 7–17 and 21–28 in helical conformation depending on the experimental environment [14], [15], [17]. Simulations on three different dimers show that hIAPP aggregation occurs by forming a helical dimer and the dimerization is mostly through the C-terminal 20–37 region containing the amyloidogenic 20–29-residue segment. Our results serve to strength previous experimental observations that the N-terminal amino acid residues 1–19 are mainly responsible for the interaction of hIAPP with membranes [30], [49] and residues 20–29 are crucial for amyloid formation [47]. It is noted that β-sheet structure is not observed in our 150-/200-ns MD simulations on dimer system as transition from α-helix to β-sheet at water-POPG lipid interface was observed experimentally within several hours [48].

By comparing the membrane perturbation extents by monomers and dimers, we find that both the number of membrane-bound hIAPP monomers and peptide-peptide interaction affect the extent of membrane perturbation. However, membrane perturbation is much more sensitive to peptide-peptide interaction. Our MD simulations demonstrate that dimeric hIAPP displays larger amplitude perturbation on POPG bilayer than the monomeric species. These results suggest that bilayer perturbation of hIAPP is cooperative and the cooperativity most likely results from the peptide-peptide interaction or peptide aggregation. Further studies are needed to understand the cooperativity mechanism of membrane perturbation by hIAPP. It is expected that hIAPP aggregation which involves multiple peptide chains would potentially cause larger membrane disruption. Our computational results provide useful insight into the nature and the atomistic details of hIAPP monomer/dimer–POPG membrane interaction and the first steps of hIAPP-induced membrane disruption.

## Supporting Information

Figure S1 **Three different dimers along with the construction process of dimer2 and dimer3 from dimer1.** Dimer2 and dimer3 are constructed by rotating each hIAPP chain in dimer1 90° around its backbones under clockwise or counter-clockwise directions. All the hIAPP chains in the three dimers have the same conformation and the only difference for the three dimers is the helix-helix interface. In all the dimers, helix is in purple and coil in green.
(TIF)

Figure S2 **Time evolution of the z-coordinate of the hIAPP peptide atom most deeply inserted in the bilayer**. The data are given for the two MD runs of 1mono and dimer1 systems. The z-axis is normal to the bilayer surface. The z-coordinate of the bilayer center is zero. Upper leaflet: z>0 nm, lower leaflet: z<0 nm.
(TIF)

Figure S3 **Membrane insertion depth of hIAPP in eight 10-ns MD runs for 1mono and dimer1 systems.** The insertion depth of hIAPP peptide is estimated by the z-position of the most deeply inserted residue in the bilayer. The z-position is averaged over the last 1 ns for each MD run. The z-coordinate of the bilayer center is zero. Upper leaflet: z>0 nm, lower leaflet: z<0 nm.
(TIF)

Figure S4 **Initial state and the simulation results of dimer2 and dimer3.** Initial state of dimer2 (A) and dimer3 (D). Final states (t = 200 ns) of dimer2 (B) and dimer3 (D); time evolution of the number of atomic contacts between two different regions (residues 1–19 and 20–37) of chain A and chain B for dimer2 (C) and dimer3 (F). In all the snapshots, peptide helical structure is in purple and coil in cyan, water molecules are in red dots, and the phosphorus atoms of lipids are in tan spheres. Residues 18–20 are in green and in vdW representation. For clarity, counterions and the other atoms of POPG lipids are not shown.
(TIF)

Figure S5 **The interaction energy of hIAPP with POPG bilayer (per lipid) in 1mono and dimer systems.** The interaction energy is calculated for peptide-lipid head group and peptide-lipid tail group. For dimer system, the interaction energies of chain A and chain B with the two lipid groups are given separately. The data are averaged over the last 50 ns for each MD run.
(TIF)

Figure S6 **Initial state and the simulation results of 2mono system in two independent MD trajectories.** Initial state (A). Final states (t = 150 ns) of the system generated in MD runs of 2mono-R1 (B) and 2mono-R2 (C); Time evolution of the z-coordinate of the hIAPP atom most deeply inserted in the bilayer for the two chains in each MD run (D). The z-coordinate of the bilayer center is zero. Snapshots in (A)∼(C) are shown by using the same representations as those used in Fig. S4.
(TIF)

## References

[1] Butterfield SM, Lashuel HA (2010). Amyloidogenic Protein Membrane Interactions: Mechanistic Insight from Model Systems.. Angew Chem Int Edit.

[2] Kayed R, Sokolov Y, Edmonds B, McIntire TM, Milton SC (2004). Permeabilization of Lipid Bilayers Is a Common Conformation-dependent Activity of Soluble Amyloid Oligomers in Protein Misfolding Diseases.. J Biol Chem.

[3] Gurlo T, Ryazantsev S, Huang CJ, Yeh MW, Reber HA (2010). Evidence for Proteotoxicity in beta Cells in Type 2 Diabetes Toxic Islet Amyloid Polypeptide Oligomers Form Intracellularly in the Secretory Pathway.. Am J Pathol.

[4] Cooper GJS, Willis AC, Clark A, Turner RC, Sim RB (1987). Purification and Characterization of a Peptide from Amyloid-Rich Pancreases of Type-2 Diabetic-Patients.. Proc Natl Acad Sci U S A.

[5] Janson J, Ashley RH, Harrison D, McIntyre S, Butler PC (1999). The mechanism of islet amyloid polypeptide toxicity is membrane disruption by intermediate-sized toxic amyloid particles.. Diabetes.

[6] Smith PE, Brender JR, Ramamoorthy A (2009). Induction of negative curvature as a mechanism of cell toxicity by amyloidogenic peptides: the case of islet amyloid polypeptide.. J Am Chem Soc.

[7] Quist A, Doudevski L, Lin H, Azimova R, Ng D (2005). Amyloid ion channels: A common structural link for protein-misfolding disease.. Proc Natl Acad Sci U S A.

[8] Engel MFM, Khemtémourian L, Kleijer CC, Meeldijk HJD, Jacobs J (2008). Membrane damage by human islet amyloid polypeptide through fibril growth at the membrane.. Proc Natl Acad Sci U S A.

[9] Knight JD, Hebda JA, Miranker AD (2006). Conserved and cooperative assembly of membrane-bound alpha-helical states of islet amyloid polypeptide.. Biochemistry.

[10] Brender JR, Salamekh S, Ramamoorthy A (2011). Membrane Disruption and Early Events in the Aggregation of the Diabetes Related Peptide IAPP from a Molecular Perspective.. Acc Chem Res.

[11] Knight JD, Miranker AD (2004). Phospholipid catalysis of diabetic amyloid assembly.. J Mol Biol.

[12] Jayasinghe SA, Langen R (2005). Lipid membranes modulate the structure of islet amyloid polypeptide.. Biochemistry.

[13] Last NB, Rhoades E, Miranker AD (2011). Islet amyloid polypeptide demonstrates a persistent capacity to disrupt membrane integrity.. Proc Natl Acad Sci U S A.

[14] Patil SM, Xu S, Sheftic SR, Alexandrescu AT (2009). Dynamic alpha-helix structure of micelle-bound human amylin.. J Biol Chem.

[15] Apostolidou M, Jayasinghe SA, Langen R (2008). Structure of alpha-helical membrane-bound human islet amyloid polypeptide and its implications for membrane-mediated misfolding.. J Biol Chem.

[16] Williamson JA, Loria JP, Miranker AD (2009). Helix stabilization precedes aqueous and bilayer-catalyzed fiber formation in islet amyloid polypeptide.. J Mol Biol.

[17] Nanga RPR, Brender JR, Vivekanandan S, Ramamoorthy A (2011). Structure and membrane orientation of IAPP in its natively amidated form at physiological pH in a membrane environment.. Biochim Biophys Acta-Biomembranes.

[18] Brender JR, Lee EL, Cavitt MA, Gafni A, Steel DG (2008). Amyloid fiber formation and membrane disruption are separate processes localized in two distinct regions of IAPP, the type-2-diabetes-related peptide.. J Am Chem Soc.

[19] Dupuis NF, Wu C, Shea JE, Bowers MT (2009). Human islet amyloid polypeptide monomers form ordered beta-hairpins: a possible direct amyloidogenic precursor.. J Am Chem Soc.

[20] Laghaei R, Mousseau N, Wei G (2010). Effect of the disulfide bond on the monomeric structure of human amylin studied by combined Hamiltonian and temperature replica exchange molecular dynamics simulations.. J Phys Chem B.

[21] Reddy AS, Wang L, Singh S, Ling YL, Buchanan L (2010). Stable and Metastable States of Human Amylin in Solution.. Biophys J.

[22] Wei L, Jiang P, Xu WX, Li H, Zhang H (2011). The Molecular Basis of Distinct Aggregation Pathways of Islet Amyloid Polypeptide.. J Biol Chem.

[23] Andrews MN, Winter R (2011). Comparing the structural properties of human and rat islet amyloid polypeptide by MD computer simulations.. Biophys Chem.

[24] Colombo G, Daidone I, Gazit E, Amadei A, Di Nola A (2005). Molecular dynamics simulation of the aggregation of the core-recognition motif of the islet amylolid polypeptide in explicit water.. Proteins.

[25] Mo Y, Lu Y, Wei G, Derreumaux P (2009). Structural diversity of the soluble trimers of the human amylin(20–29) peptide revealed by molecular dynamics simulations.. J Chem Phys.

[26] Rivera E, Straub J, Thirumalai D (2009). Sequence and Crowding Effects in the Aggregation of a 10-Residue Fragment Derived from Islet Amyloid Polypeptide.. Biophys J.

[27] Jiang P, Xu W, Mu Y (2009). Amyloidogenesis abolished by proline substitutions but enhanced by lipid binding.. PLoS Comput Biol.

[28] Sciacca MFM, Pappalardo M, Attanasio F, Milardi D, La Rosa C (2010). Are fibril growth and membrane damage linked processes? An experimental and computational study of IAPP(12–18) and IAPP(21–27) peptides.. New J Chem.

[29] Elmore DE (2006). Molecular dynamics simulation of a phosphatidylglycerol membrane.. FEBS Lett.

[30] Engel MF, Yigittop H, Elgersma RC, Rijkers DT, Liskamp RM (2006). Islet amyloid polypeptide inserts into phospholipid monolayers as monomer.. J Mol Biol.

[31] Jaud S, Fernandez-Vidal M, Nilsson I, Meindi-Beinker NM, Hubner NC (2009). Insertion of short transmembrane helices by the Sec61 translocon.. Proc Natl Acad Sci U S A.

[32] Chang ZW, Luo Y, Zhang Y, Wei GH (2011). Interactions of A beta 25–35 beta-Barrel-like Oligomers with Anionic Lipid Bilayer and Resulting Membrane Leakage: An All-Atom Molecular Dynamics Study.. J Phys Chem B.

[33] Kandt C, Ash WL, Tieleman DP (2007). Setting up and running molecular dynamics simulations of membrane proteins.. Methods.

[34] Lindahl E, Hess B, van der Spoel D (2001). GROMACS 3.0: a package for molecular simulation and trajectory analysis.. J Mol Model.

[35] Berger O, Edholm O, Jahnig F (1997). Molecular dynamics simulations of a fluid bilayer of dipalmitoylphosphatidylcholine at full hydration, constant pressure, and constant temperature.. Biophys J.

[36] van Gunsteren WF, Berendsen HJC (1987).

[37] Berendsen HJC, Postma JPM, Gunsteren WFv, Hermans J (1981). Interaction models for water in relation to protein hydration.. D Reidel Publishing Co.

[38] Hess B, Bekker H, Berendsen HJC, Fraaije JGEM (1997). LINCS: A linear constraint solver for molecular simulations.. J Comput Chem.

[39] Miyamoto S, Kollman PA (1992). Settle - an Analytical Version of the Shake and Rattle Algorithm for Rigid Water Models.. J Comput Chem.

[40] Darden T, York D, Pedersen L (1993). Particle Mesh Ewald - an N.Log(N) Method for Ewald Sums in Large Systems.. J Chem Phys.

[41] Patra M, Karttunen M, Hyvonen MT, Falck E, Lindqvist P (2003). Molecular dynamics simulations of lipid bilayers: Major artifacts due to truncating electrostatic interactions.. Biophys J.

[42] Wiedmann T, Salmon A, Wong V (1993). Phase-Behavior of Mixtures of Dppc and Popg.. Biochim Biophys Acta.

[43] Tolokh IS, Vivcharuk V, Tomberli B, Gray CG (2009). Binding free energy and counterion release for adsorption of the antimicrobial peptide lactoferricin B on a POPG membrane.. Phys Rev E.

[44] Berendsen HJC, Postma JPM, van Gunsteren WF, DiNola A, Haak JR (1984). Molecular dynamics with coupling to an external bath.. J Chem Phys.

[45] Kabsch W, Sander C (1983). Dictionary of Protein Secondary Structure - Pattern-Recognition of Hydrogen-Bonded and Geometrical Features.. Biopolymers.

[46] Humphrey W, Dalke A, Schulten K (1996). VMD: Visual molecular dynamics.. J Mol Graph.

[47] Goldsbury C, Goldie K, Pellaud J, Seelig J, Frey P (2000). Amyloid Fibril Formation from Full-Length and Fragments of Amylin.. J Struct Biol.

[48] Lopes DHJ, Meister A, Gohlke A, Hauser A, Blume A (2007). Mechanism of islet amyloid polypeptide fibrillation at lipid interfaces studied by infrared reflection absorption spectroscopy.. Biophys J.

[49] Jayasinghe SA, Langen R (2007). Membrane interaction of islet amyloid polypeptide.. Biochim Biophys Acta-Biomembranes.

[50] Vermeer LS, de Groot BL, Reat V, Milon A, Czaplicki J (2007). Acyl chain order parameter profiles in phospholipid bilayers: computation from molecular dynamics simulations and comparison with H-2 NMR experiments.. Eur Biophys J Biophy.

[51] Haataja L, Gurlo T, Huang CJ, Butler PC (2008). Islet amyloid in type 2 diabetes, and the toxic oligomer hypothesis.. Endocr Rev.

[52] Simakova O, Arispe NJ (2007). The Cell-Selective Neurotoxicity of the Alzheimer's Aβ Peptide Is Determined by Surface Phosphatidylserine and Cytosolic ATP Levels. Membrane Binding Is Required for Aβ Toxicity.. J Neurosci.

[53] Jao CC, Der-Sarkissian A, Chen J, Langen R (2004). Structure of membrane-bound α-synuclein studied by site-directed spin labeling.. Proc Natl Acad Sci U S A.

[54] Yu X, Zheng J (2011). Cholesterol Promotes the Interaction of Alzheimer β-Amyloid Monomer with Lipid Bilayer.. J Mol Biol.

