# Supplementary figures and images for: Lipid Interaction and Membrane Perturbation of Human Islet Amyloid Polypeptide Monomer and Dimer by Molecular Dynamics Simulations
Source: PLoS One. 2012 May 31;7(5):e38191. doi: 10.1371/journal.pone.0038191 (PMC3364971; doi:10.1371/journal.pone.0038191)

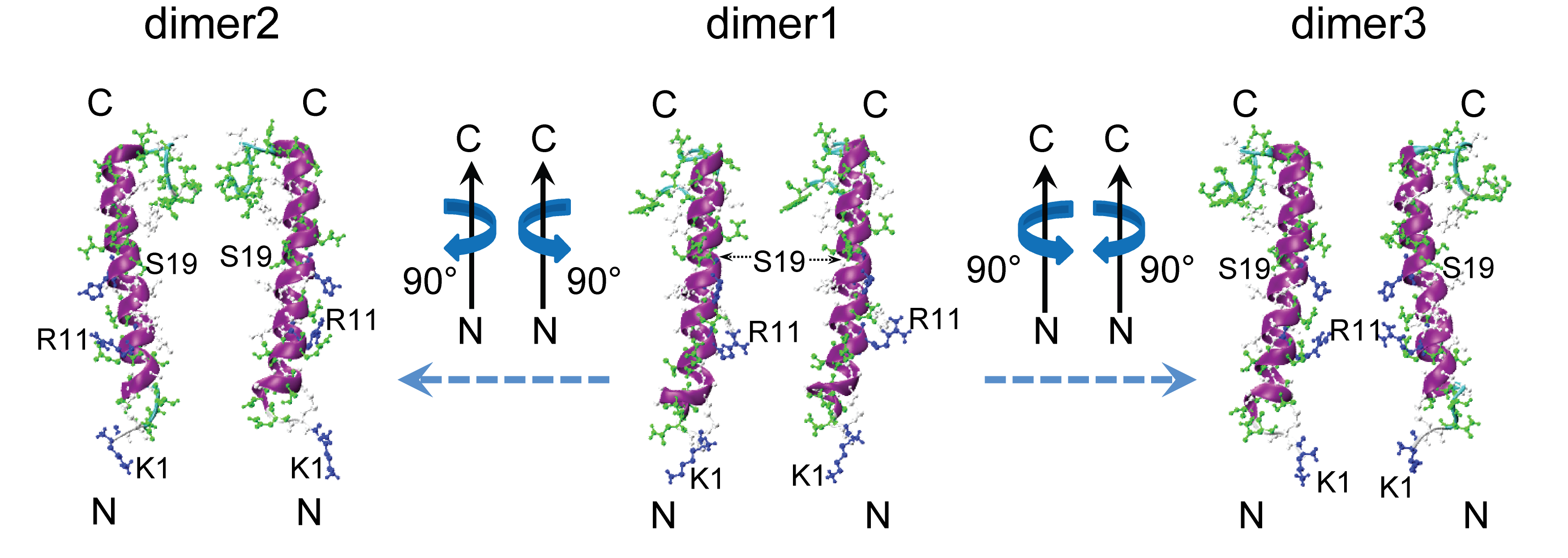

Supplement: Figure S1 — Three different dimers along with the construction process of dimer2 and dimer3 from dimer1. Dimer2 and dimer3 are constructed by rotating each hIAPP chain in dimer1 90° around its backbones under clockwise or counter-clockwise directions. All the hIAPP chains in the three dimers have the same conformation and the only difference for the three dimers is the helix-helix interface. In all the dimers, helix is in purple and coil in green. (TIF) [file pone.0038191.s001.tif]

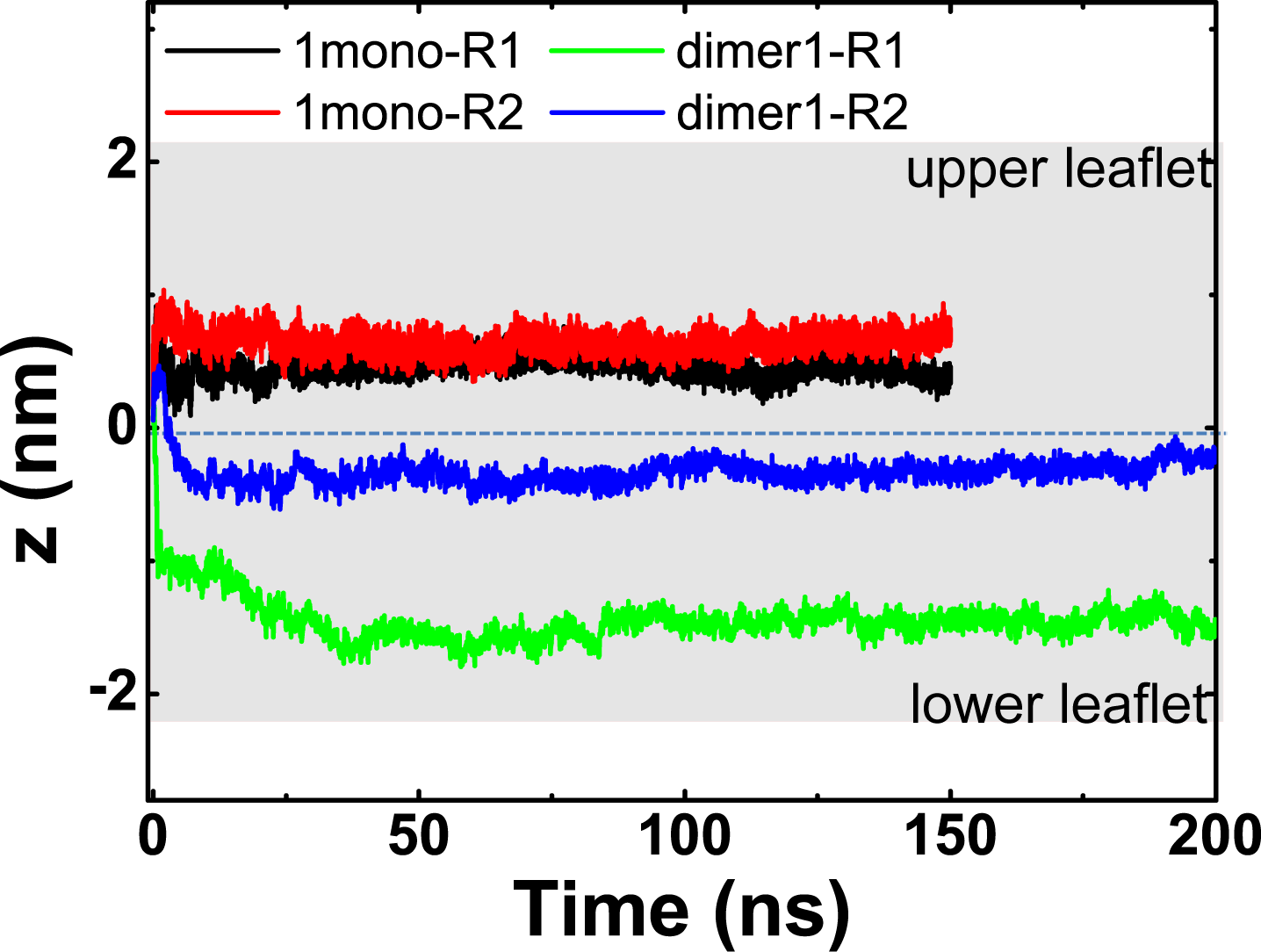

Supplement: Figure S2 — Time evolution of the z-coordinate of the hIAPP peptide atom most deeply inserted in the bilayer. The data are given for the two MD runs of 1mono and dimer1 systems. The z-axis is normal to the bilayer surface. The z-coordinate of the bilayer center is zero. Upper leaflet: z>0 nm, lower leaflet: z<0 nm. (TIF) [file pone.0038191.s002.tif]

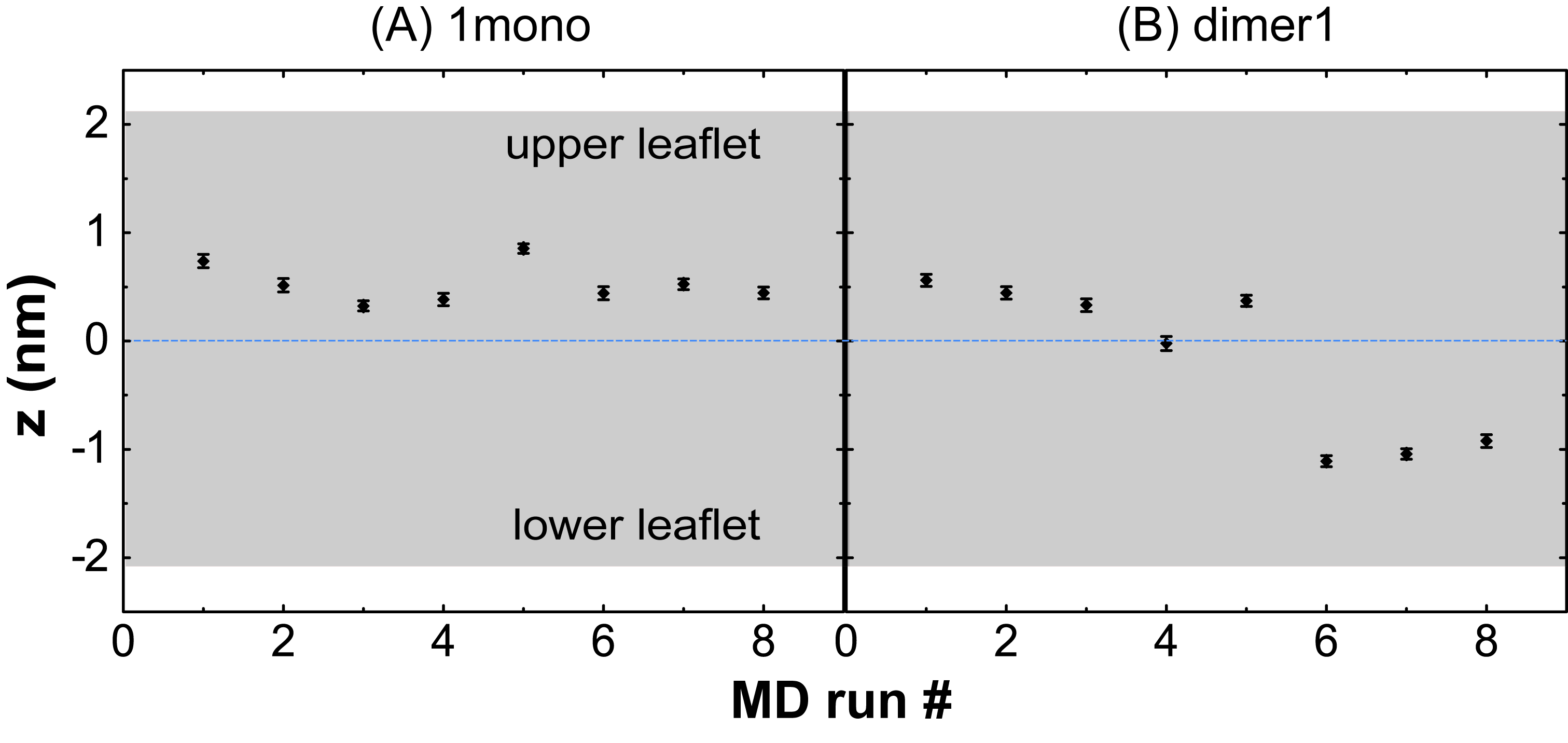

Supplement: Figure S3 — Membrane insertion depth of hIAPP in eight 10-ns MD runs for 1mono and dimer1 systems. The insertion depth of hIAPP peptide is estimated by the z-position of the most deeply inserted residue in the bilayer. The z-position is averaged over the last 1 ns for each MD run. The z-coordinate of the bilayer center is zero. Upper leaflet: z>0 nm, lower leaflet: z<0 nm. (TIF) [file pone.0038191.s003.tif]

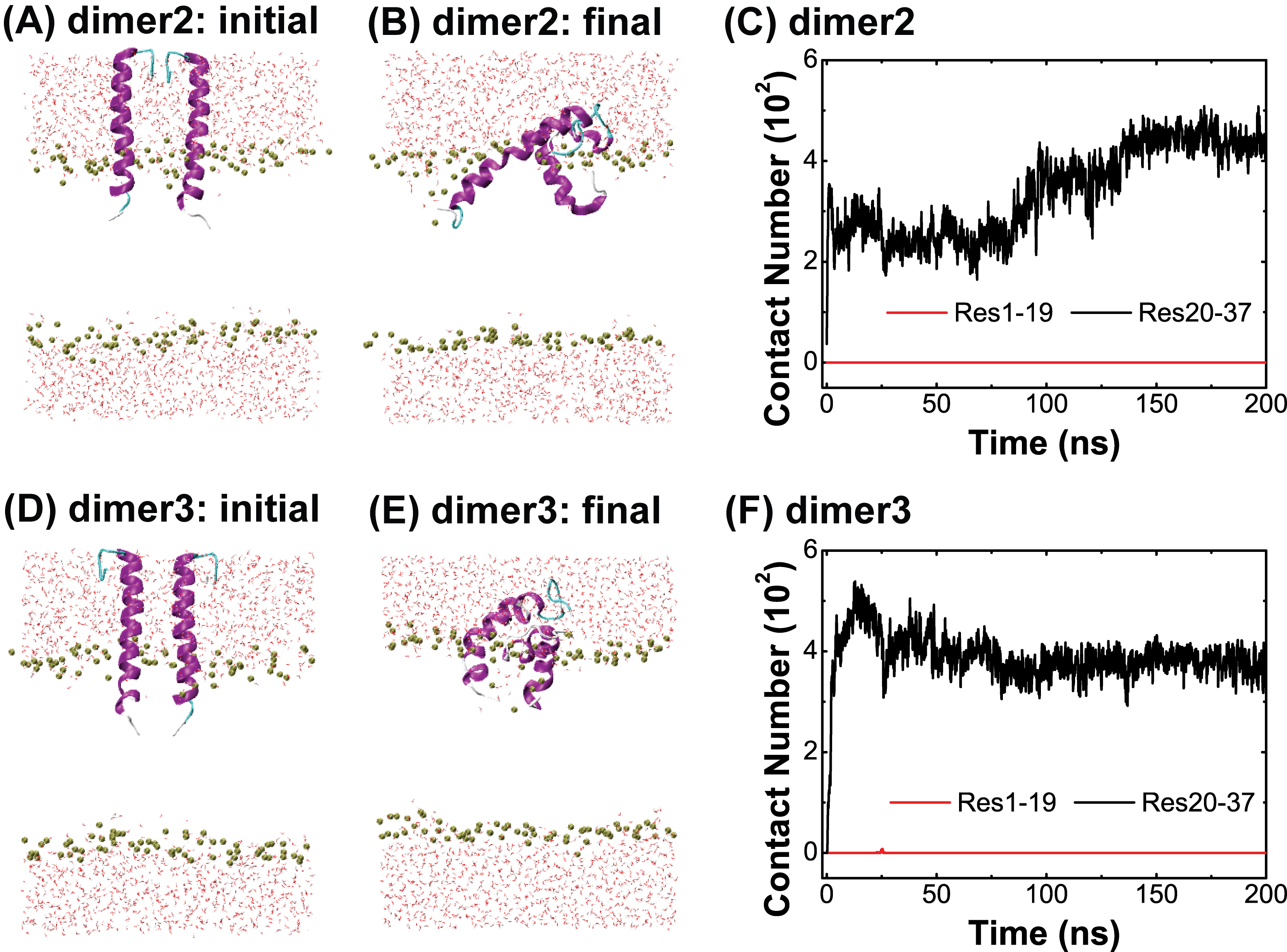

Supplement: Figure S4 — Initial state and the simulation results of dimer2 and dimer3. Initial state of dimer2 (A) and dimer3 (D). Final states (t = 200 ns) of dimer2 (B) and dimer3 (D); time evolution of the number of atomic contacts between two different regions (residues 1–19 and 20–37) of chain A and chain B for dimer2 (C) and dimer3 (F). In all the snapshots, peptide helical structure is in purple and coil in cyan, water molecules are in red dots, and the phosphorus atoms of lipids are in tan spheres. Residues 18–20 are in green and in vdW representation. For clarity, counterions and the other atoms of POPG lipids are not shown. (TIF) [file pone.0038191.s004.tif]

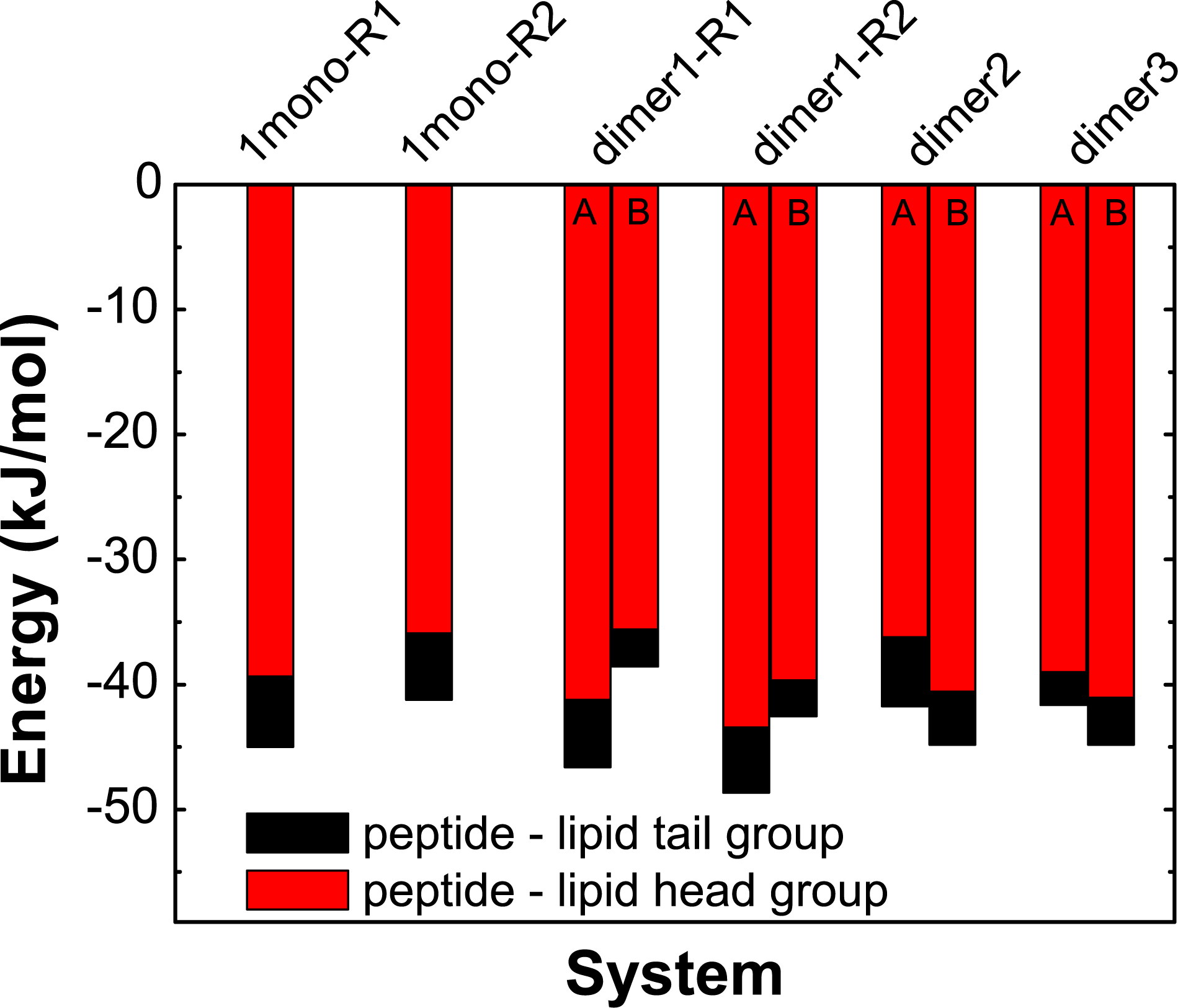

Supplement: Figure S5 — The interaction energy of hIAPP with POPG bilayer (per lipid) in 1mono and dimer systems. The interaction energy is calculated for peptide-lipid head group and peptide-lipid tail group. For dimer system, the interaction energies of chain A and chain B with the two lipid groups are given separately. The data are averaged over the last 50 ns for each MD run. (TIF) [file pone.0038191.s005.tif]

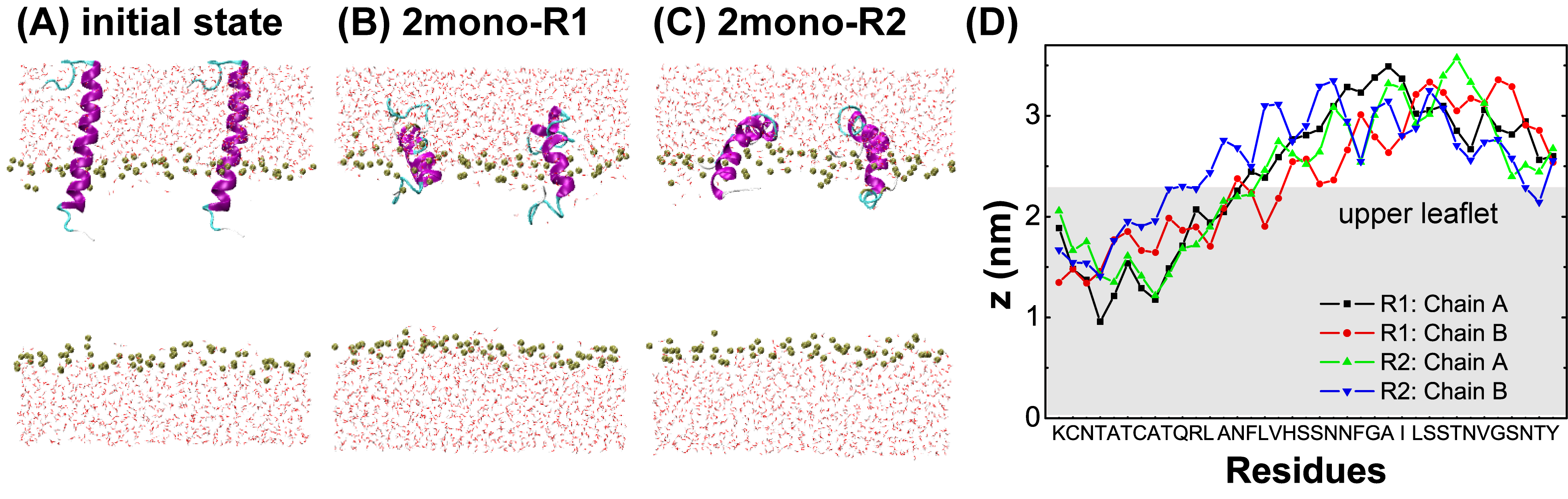

Supplement: Figure S6 — Initial state and the simulation results of 2mono system in two independent MD trajectories. Initial state (A). Final states (t = 150 ns) of the system generated in MD runs of 2mono-R1 (B) and 2mono-R2 (C); Time evolution of the z-coordinate of the hIAPP atom most deeply inserted in the bilayer for the two chains in each MD run (D). The z-coordinate of the bilayer center is zero. Snapshots in (A)∼(C) are shown by using the same representations as those used in Fig. S4. (TIF) [file pone.0038191.s006.tif]
